# Bis(*N*-triisopropyl­silyl­quinolin-8-aminato)nickel(II)

**DOI:** 10.1107/S1600536809051708

**Published:** 2009-12-04

**Authors:** Astrid Malassa, Benjamin Jäger, Helmar Görls, Matthias Westerhausen

**Affiliations:** a Institute of Inorganic and Analytical Chemistry, Friedrich-Schiller-Universität Jena, August-Bebel-Str. 2, D-07743 Jena, Germany

## Abstract

The reddish-brown title complex, [Ni(C_18_H_27_N_2_Si)_2_], was prepared *via* the salt-metathesis reaction of *N*-triisopropyl­silyl-8-amido­quinoline lithium with nickelocene (NiCp_2_). The asymmetric unit contains two symmetry-independent mol­ecules with the Ni atoms in distorted tetra­hedral environments.

## Related literature

The reaction of *N*-trialkyl­silyl-8-amido­quinoline lithium (Jonas *et al.*, 2000 ▶) with nickelocene yields paramagnetic bis­(*N*-trialkyl­silyl-8-amido­quinoline)nickel(II) (Lee *et al.*, 2000 ▶). The isostructural zinc and magnesium derivatives are thermally stable whereas bis­(*N*-trimethyl­silyl-8-amido­quinoline) cadmium(II) liberates dimethyl­cadmium at 513 K (Englehardt *et al.*, 1991 ▶). Transamination of Zn[N(SiMe_3_)_2_]_2_ with *N*-trialkyl­silyl-8-amino­quinoline gives heteroleptic *N*-trialkyl­silyl-8-amido­quinoline (Malassa *et al.*, 2008 ▶). In contrast to these 8-amido­quinoline complexes, neutral 8-amino­quinoline can easily act as a bidentate base to metal cations, see: Engelter *et al.* (1989 ▶); Fanning & Taylor (1965 ▶); Nast *et al.* (1961 ▶); Nielsen & Dahl (1966 ▶).
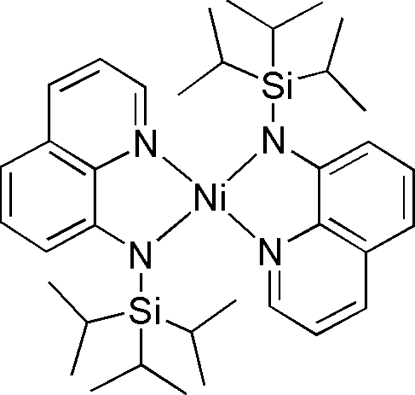


## Experimental

### 

#### Crystal data

[Ni(C_18_H_27_N_2_Si)_2_]
*M* *_r_* = 657.72
Triclinic, 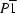

*a* = 12.3062 (5) Å
*b* = 17.7015 (5) Å
*c* = 18.4194 (6) Å
α = 68.956 (2)°
β = 72.025 (2)°
γ = 85.540 (2)°
*V* = 3559.6 (2) Å^3^
*Z* = 4
Mo *K*α radiation
μ = 0.64 mm^−1^
*T* = 183 K
0.04 × 0.04 × 0.04 mm

#### Data collection

Nonius KappaCCD diffractometer
25470 measured reflections
15935 independent reflections
12031 reflections with *I* > 2σ(*I*)
*R*_int_ = 0.034

#### Refinement

*R*[*F* ^2^ > 2σ(*F* ^2^)] = 0.047
*wR*(*F* ^2^) = 0.123
*S* = 1.02
15935 reflections
799 parameters
H-atom parameters constrained
Δρ_max_ = 0.50 e Å^−3^
Δρ_min_ = −0.44 e Å^−3^

### 

Data collection: *COLLECT* (Nonius, 1998 ▶); cell refinement: *DENZO* (Otwinowski & Minor, 1997 ▶); data reduction: *DENZO*; program(s) used to solve structure: *SHELXS97* (Sheldrick, 2008 ▶); program(s) used to refine structure: *SHELXL97* (Sheldrick, 2008 ▶); molecular graphics: *SHELXTL/PC* (Sheldrick, 2008 ▶); software used to prepare material for publication: *SHELXL97*.

## Supplementary Material

Crystal structure: contains datablocks I, global. DOI: 10.1107/S1600536809051708/jh2120sup1.cif

Structure factors: contains datablocks I. DOI: 10.1107/S1600536809051708/jh2120Isup2.hkl

Additional supplementary materials:  crystallographic information; 3D view; checkCIF report

## Figures and Tables

**Fig. 1.**
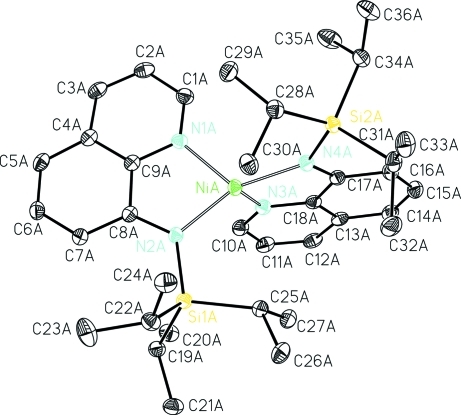
Molecular structure of I. Displacement ellipsoids are drawn at the 40% probability level. H atoms have been omitted for clarity.

## supplementary crystallographic information

### Comment

The reaction of *N*-trialkylsilyl-8-amidoquinoline lithium (Jonas *et al.* 2000) with nickelocene yields paramagnetic bis(*N*-trialkylsilyl-8-amidoquinoline)nickel(II) (Lee *et al.* 2000) (Si*R*_3_ = SiMe_3_, SiMe_2_tBu) with the metal centers in distorted tetrahedral environments. Isostructural zinc and magnesium derivatives are thermally stable whereas bis(*N*-trimethylsilyl-8-amidoquinoline)cadmium(II) liberates dimethylcadmium at 240°C (Englehardt *et al.* 1991). Transamination of Zn[N(SiMe_3_)_2_]_2_ with *N*-trialkylsilyl-8-aminoquinoline gives heteroleptic *N*-trialkylsilyl-8-amidoquinoline zinc(II)bis(trimethylsilyl)amide (Malassa *et al.* 2008). The metallation of 8-aminoquinoline with [(tmeda)NiMe_2_] yields bis(8-amidoquinoline) nickel(II) with a square planar coordinated nickel center. In contrast to these rather rare examples of 8-amidoquinoline complexes, neutral 8-aminoquinoline can easily act as a bidentate base to metal cations (see *e.g.* Engelter *et al.* 1989, Fanning *et al.* 1965, Nast *et al.* 1961, Nielsen *et al.* 1966).

The reaction of nickelocene with *N*-triisopropylsilyl-8-amidoquinoline lithium yields the reddish-brown title complex bis(*N*-triisopropylsilyl-8-amidoquinoline) nickel(II). This compound crystallizes from diethyl ether with two crystallographically independent molecules with similar configurations. The nickel atoms are in distorted tetrahedral environments despite the fact that amido and pyridyl bases have to be considered as strong Lewis bases because the bulky triisopropylsilyl groups prevent a distorted square planar coordination sphere of the Ni centers. Due to an additional electrostatic attraction the Ni—N_amido_ bond lengths (av. value 196.4 pm) are smaller than the Ni—N_pyridyl_ distances with an average value of 200.3 pm.

### Experimental

All manipulations were carried out by using modified Schlenk techniques under an atmosphere of argon. Prior to use, THF and diethyl ether were distilled over sodium/benzophenone.

*N*-Triisopropylsilyl-8-aminochinolin (0.46 g, 1.54 mmol), dissolved at -78°C in 10 ml of THF, was lithiated with 1 mL of a 1,6 *M* solution of *n*BuLi in hexane. To this solution 0.15 g of [NiCp_2_] (1.54 mmol) in 13 ml of THF was added drop-wise while a temperature of -78°C was maintained in the reaction flask. The cooling bath was removed and the red-brown reaction mixture stirred for additional 12 h. Thereafter, all volatiles were removed in vacuum and the residue dissolved in 20 ml of diethyl ether. After filtration the volume of the mother liquor was reduced to half of the original volume. At -20°C reddish brown crystals precipitated. Yield: 0.10 g (0.15 mmol, 21%). IR (Nujol, KBr windows, cm^-1^): 1562 m,1508 m, 1496 m, 1365 m, 1315 s, 1280 m, 1261 m, 1224 w, 1115 m, 1096 m, 1015 m,934 m, 913 w, 882 m, 819 s, 790 m, 757 m, 739 m, 673 w, 648 w, 621 w, 588 w,561 w, 532 w, 500 w. MS (DEI (%)): m/*z* = 656 (*M*^+^, 5), 614 ([*M* - *i*Pr]^+^, 4), 300 (22), 257 (100), 171 (58). Elemental analysis (C_36_H_54_N_4_NiSi_2_; 657,71 g mol^-1^): calcd. C 65.74, H 8.28, N 8.52; found C 62.82, H 9.00, N 7.48.

### Refinement

All hydrogen atoms were set to idealized positions and refined with 1.2 times (1.5 for methyl groups) the isotropic displacement parameter of the corresponding carbon atom.

### Crystal data

**Table:**

[Ni(C_18_H_27_N_2_Si)_2_]	*Z* = 4
*M**_r_* = 657.72	*F*(000) = 1416
Triclinic, *P*1	*D*_x_ = 1.227 Mg m^−^^3^
Hall symbol: -P 1	Mo *K*α radiation, λ = 0.71073 Å
*a* = 12.3062 (5) Å	Cell parameters from 25470 reflections
*b* = 17.7015 (5) Å	θ = 2.0–27.5°
*c* = 18.4194 (6) Å	µ = 0.64 mm^−^^1^
α = 68.956 (2)°	*T* = 183 K
β = 72.025 (2)°	Prism, brown
γ = 85.540 (2)°	0.04 × 0.04 × 0.04 mm
*V* = 3559.6 (2) Å^3^	

### Data collection

**Table:**

Nonius KappaCCD diffractometer	12031 reflections with *I* > 2σ(*I*)
Radiation source: fine-focus sealed tube	*R*_int_ = 0.034
graphite	θ_max_ = 27.5°, θ_min_ = 2.0°
phi– + ω–scan	*h* = −15→14
25470 measured reflections	*k* = −22→21
15935 independent reflections	*l* = −23→21

### Refinement

**Table:**

Refinement on *F*^2^	Primary atom site location: structure-invariant direct methods
Least-squares matrix: full	Secondary atom site location: difference Fourier map
*R*[*F*^2^ > 2σ(*F*^2^)] = 0.047	Hydrogen site location: inferred from neighbouring sites
*wR*(*F*^2^) = 0.123	H-atom parameters constrained
*S* = 1.02	*w* = 1/[σ^2^(*F*_o_^2^) + (0.0507*P*)^2^ + 2.9757*P*] where *P* = (*F*_o_^2^ + 2*F*_c_^2^)/3
15935 reflections	(Δ/σ)_max_ = 0.001
799 parameters	Δρ_max_ = 0.50 e Å^−^^3^
0 restraints	Δρ_min_ = −0.44 e Å^−^^3^

### Special details

**Table:**

Geometry. All e.s.d.'s (except the e.s.d. in the dihedral angle between two l.s. planes) are estimated using the full covariance matrix. The cell e.s.d.'s are taken into account individually in the estimation of e.s.d.'s in distances, angles and torsion angles; correlations between e.s.d.'s in cell parameters are only used when they are defined by crystal symmetry. An approximate (isotropic) treatment of cell e.s.d.'s is used for estimating e.s.d.'s involving l.s. planes.
Refinement. Refinement of *F*^2^ against ALL reflections. The weighted *R*-factor *wR* and goodness of fit *S* are based on *F*^2^, conventional *R*-factors *R* are based on *F*, with *F* set to zero for negative *F*^2^. The threshold expression of *F*^2^ > σ(*F*^2^) is used only for calculating *R*-factors(gt) *etc*. and is not relevant to the choice of reflections for refinement. *R*-factors based on *F*^2^ are statistically about twice as large as those based on *F*, and *R*- factors based on ALL data will be even larger.

### Fractional atomic coordinates and isotropic or equivalent isotropic displacement parameters (Å^2^)

**Table:**

	*x*	*y*	*z*	*U*_iso_*/*U*_eq_	
Ni1A	0.22671 (3)	0.404287 (18)	0.365455 (19)	0.02670 (8)	
Si1A	0.31556 (6)	0.30222 (4)	0.52291 (4)	0.02685 (15)	
Si2A	0.28061 (6)	0.60410 (4)	0.27705 (4)	0.02624 (15)	
N1A	0.06609 (18)	0.36971 (12)	0.38461 (13)	0.0289 (4)	
N2A	0.20579 (17)	0.32527 (12)	0.47700 (12)	0.0262 (4)	
N3A	0.30614 (18)	0.35165 (12)	0.28448 (13)	0.0294 (5)	
N4A	0.30124 (18)	0.50415 (12)	0.27932 (12)	0.0270 (4)	
C1A	0.0024 (2)	0.39528 (16)	0.33440 (17)	0.0353 (6)	
H1AA	0.0335	0.4361	0.2825	0.042*	
C2A	−0.1088 (2)	0.36428 (18)	0.35519 (18)	0.0394 (6)	
H2AA	−0.1527	0.3838	0.3183	0.047*	
C3A	−0.1527 (2)	0.30520 (17)	0.42981 (18)	0.0360 (6)	
H3AA	−0.2282	0.2840	0.4451	0.043*	
C4A	−0.0872 (2)	0.27538 (15)	0.48434 (16)	0.0296 (5)	
C5A	−0.1253 (2)	0.21290 (16)	0.56120 (17)	0.0338 (6)	
H5AA	−0.1997	0.1886	0.5800	0.041*	
C6A	−0.0529 (2)	0.18804 (16)	0.60827 (17)	0.0347 (6)	
H6AA	−0.0780	0.1456	0.6597	0.042*	
C7A	0.0574 (2)	0.22324 (15)	0.58293 (16)	0.0314 (5)	
H7AA	0.1040	0.2037	0.6180	0.038*	
C8A	0.1011 (2)	0.28572 (14)	0.50834 (15)	0.0266 (5)	
C9A	0.0240 (2)	0.31006 (14)	0.45882 (15)	0.0270 (5)	
C10A	0.3044 (2)	0.27422 (16)	0.29294 (18)	0.0352 (6)	
H10A	0.2504	0.2379	0.3391	0.042*	
C11A	0.3797 (2)	0.24414 (16)	0.23586 (19)	0.0378 (6)	
H11A	0.3772	0.1882	0.2436	0.045*	
C12A	0.4564 (2)	0.29635 (17)	0.16916 (18)	0.0368 (6)	
H12A	0.5082	0.2765	0.1304	0.044*	
C13A	0.4596 (2)	0.38015 (16)	0.15709 (16)	0.0311 (5)	
C14A	0.5352 (2)	0.43815 (17)	0.08965 (16)	0.0358 (6)	
H14A	0.5890	0.4227	0.0483	0.043*	
C15A	0.5295 (2)	0.51736 (17)	0.08480 (16)	0.0365 (6)	
H15A	0.5798	0.5568	0.0390	0.044*	
C16A	0.4522 (2)	0.54227 (16)	0.14508 (16)	0.0328 (6)	
H16A	0.4513	0.5980	0.1385	0.039*	
C17A	0.3765 (2)	0.48775 (15)	0.21454 (15)	0.0273 (5)	
C18A	0.3817 (2)	0.40518 (15)	0.21780 (15)	0.0275 (5)	
C19A	0.3501 (2)	0.19049 (15)	0.55095 (16)	0.0312 (5)	
H19A	0.2868	0.1608	0.6007	0.037*	
C20A	0.3507 (3)	0.15513 (17)	0.48601 (19)	0.0406 (7)	
H20A	0.3658	0.0972	0.5053	0.061*	
H20B	0.4104	0.1832	0.4352	0.061*	
H20C	0.2761	0.1624	0.4761	0.061*	
C21A	0.4609 (2)	0.17276 (17)	0.57561 (19)	0.0396 (6)	
H21A	0.4732	0.1144	0.5922	0.059*	
H21B	0.4541	0.1908	0.6212	0.059*	
H21C	0.5257	0.2018	0.5291	0.059*	
C22A	0.2903 (2)	0.32499 (15)	0.62029 (16)	0.0330 (6)	
H22A	0.3671	0.3209	0.6291	0.040*	
C23A	0.2123 (3)	0.2658 (2)	0.69940 (18)	0.0531 (8)	
H23A	0.2176	0.2784	0.7459	0.080*	
H23B	0.2361	0.2104	0.7053	0.080*	
H23C	0.1331	0.2709	0.6977	0.080*	
C24A	0.2552 (3)	0.41099 (17)	0.61376 (18)	0.0388 (6)	
H24A	0.2546	0.4199	0.6634	0.058*	
H24B	0.1786	0.4189	0.6072	0.058*	
H24C	0.3098	0.4496	0.5664	0.058*	
C25A	0.4425 (2)	0.37000 (15)	0.44380 (16)	0.0315 (6)	
H25A	0.4095	0.4218	0.4161	0.038*	
C26A	0.5281 (2)	0.39364 (18)	0.47874 (19)	0.0414 (7)	
H26A	0.5888	0.4298	0.4340	0.062*	
H26B	0.5618	0.3447	0.5081	0.062*	
H26C	0.4882	0.4215	0.5163	0.062*	
C27A	0.5117 (2)	0.33914 (18)	0.37565 (17)	0.0394 (6)	
H27A	0.5618	0.3832	0.3314	0.059*	
H27B	0.4594	0.3202	0.3545	0.059*	
H27C	0.5582	0.2943	0.3975	0.059*	
C28A	0.1732 (2)	0.60344 (15)	0.37508 (16)	0.0308 (5)	
H28A	0.1690	0.6609	0.3727	0.037*	
C29A	0.0512 (2)	0.57628 (17)	0.38801 (19)	0.0397 (6)	
H29A	−0.0001	0.5831	0.4376	0.059*	
H29B	0.0499	0.5192	0.3936	0.059*	
H29C	0.0260	0.6093	0.3411	0.059*	
C30A	0.2114 (3)	0.55495 (16)	0.45009 (16)	0.0363 (6)	
H30A	0.1577	0.5623	0.4987	0.054*	
H30B	0.2881	0.5742	0.4425	0.054*	
H30C	0.2127	0.4974	0.4570	0.054*	
C31A	0.4195 (2)	0.65577 (15)	0.26385 (16)	0.0329 (6)	
H31A	0.4548	0.6828	0.2037	0.040*	
C32A	0.5084 (3)	0.59713 (19)	0.2939 (2)	0.0444 (7)	
H32A	0.5813	0.6268	0.2767	0.067*	
H32B	0.5190	0.5543	0.2705	0.067*	
H32C	0.4816	0.5729	0.3535	0.067*	
C33A	0.4021 (3)	0.72383 (17)	0.29984 (19)	0.0431 (7)	
H33A	0.4757	0.7518	0.2851	0.065*	
H33B	0.3710	0.7004	0.3594	0.065*	
H33C	0.3487	0.7625	0.2779	0.065*	
C34A	0.2277 (2)	0.66770 (15)	0.18740 (16)	0.0340 (6)	
H34A	0.2969	0.6860	0.1386	0.041*	
C35A	0.1496 (3)	0.61987 (18)	0.1671 (2)	0.0505 (8)	
H35A	0.1339	0.6536	0.1164	0.076*	
H35B	0.0776	0.6045	0.2116	0.076*	
H35C	0.1875	0.5710	0.1604	0.076*	
C36A	0.1695 (3)	0.74443 (17)	0.1978 (2)	0.0462 (7)	
H36A	0.1457	0.7752	0.1496	0.069*	
H36B	0.2232	0.7778	0.2043	0.069*	
H36C	0.1022	0.7294	0.2463	0.069*	
Ni1B	0.05709 (3)	0.186465 (18)	0.17978 (2)	0.02737 (9)	
Si1B	−0.21235 (6)	0.14247 (4)	0.21518 (4)	0.02792 (15)	
Si2B	0.23432 (6)	0.11779 (4)	0.04677 (4)	0.02724 (15)	
N1B	0.08118 (18)	0.30640 (12)	0.14648 (13)	0.0299 (5)	
N2B	−0.10400 (18)	0.21276 (12)	0.19249 (13)	0.0277 (4)	
N3B	0.09058 (18)	0.14248 (12)	0.28678 (13)	0.0286 (4)	
N4B	0.18624 (17)	0.12248 (12)	0.14461 (13)	0.0269 (4)	
C1B	0.1775 (2)	0.34911 (17)	0.12183 (17)	0.0365 (6)	
H1BA	0.2467	0.3212	0.1213	0.044*	
C2B	0.1822 (3)	0.43342 (17)	0.09647 (18)	0.0408 (7)	
H2BA	0.2531	0.4622	0.0789	0.049*	
C3B	0.0829 (3)	0.47395 (16)	0.09732 (18)	0.0393 (6)	
H3BA	0.0850	0.5315	0.0784	0.047*	
C4B	−0.0228 (2)	0.43081 (15)	0.12615 (16)	0.0323 (6)	
C5B	−0.1300 (3)	0.46732 (16)	0.13364 (19)	0.0397 (7)	
H5BA	−0.1348	0.5245	0.1180	0.048*	
C6B	−0.2269 (2)	0.41879 (16)	0.16390 (19)	0.0386 (6)	
H6BA	−0.2991	0.4432	0.1705	0.046*	
C7B	−0.2234 (2)	0.33404 (15)	0.18565 (18)	0.0339 (6)	
H7BA	−0.2932	0.3031	0.2069	0.041*	
C8B	−0.1200 (2)	0.29407 (14)	0.17684 (15)	0.0286 (5)	
C9B	−0.0194 (2)	0.34539 (15)	0.14931 (15)	0.0285 (5)	
C10B	0.0365 (2)	0.15432 (17)	0.35555 (17)	0.0359 (6)	
H10B	−0.0224	0.1923	0.3557	0.043*	
C11B	0.0626 (3)	0.11292 (17)	0.42832 (17)	0.0390 (6)	
H11B	0.0213	0.1223	0.4770	0.047*	
C12B	0.1483 (3)	0.05889 (17)	0.42846 (17)	0.0382 (6)	
H12B	0.1664	0.0302	0.4776	0.046*	
C13B	0.2098 (2)	0.04562 (15)	0.35589 (16)	0.0313 (5)	
C14B	0.3022 (2)	−0.00709 (17)	0.34931 (18)	0.0380 (6)	
H14B	0.3256	−0.0378	0.3957	0.046*	
C15B	0.3568 (2)	−0.01270 (17)	0.27480 (19)	0.0407 (7)	
H15B	0.4202	−0.0468	0.2701	0.049*	
C16B	0.3239 (2)	0.02947 (16)	0.20487 (17)	0.0335 (6)	
H16B	0.3657	0.0233	0.1547	0.040*	
C17B	0.2307 (2)	0.08064 (14)	0.20711 (15)	0.0268 (5)	
C18B	0.1770 (2)	0.08895 (14)	0.28493 (15)	0.0274 (5)	
C19B	−0.3310 (2)	0.13621 (16)	0.31244 (17)	0.0349 (6)	
H19B	−0.3836	0.1809	0.2968	0.042*	
C20B	−0.2851 (3)	0.1516 (2)	0.37487 (19)	0.0461 (7)	
H20D	−0.3490	0.1509	0.4227	0.069*	
H20E	−0.2445	0.2045	0.3500	0.069*	
H20F	−0.2324	0.1092	0.3917	0.069*	
C21B	−0.4032 (3)	0.05698 (18)	0.3521 (2)	0.0460 (7)	
H21D	−0.4649	0.0591	0.4000	0.069*	
H21E	−0.3549	0.0114	0.3690	0.069*	
H21F	−0.4361	0.0498	0.3128	0.069*	
C22B	−0.2766 (2)	0.17488 (17)	0.12737 (17)	0.0359 (6)	
H22B	−0.3041	0.2307	0.1225	0.043*	
C23B	−0.1873 (3)	0.1844 (2)	0.04511 (19)	0.0522 (8)	
H23D	−0.2228	0.2071	0.0013	0.078*	
H23E	−0.1575	0.1313	0.0455	0.078*	
H23F	−0.1244	0.2208	0.0359	0.078*	
C24B	−0.3821 (3)	0.12528 (19)	0.1391 (2)	0.0438 (7)	
H24D	−0.4119	0.1505	0.0926	0.066*	
H24E	−0.4411	0.1239	0.1895	0.066*	
H24F	−0.3603	0.0699	0.1426	0.066*	
C25B	−0.1370 (2)	0.04357 (15)	0.22502 (18)	0.0364 (6)	
H25B	−0.0618	0.0585	0.1813	0.044*	
C26B	−0.1063 (3)	0.00331 (17)	0.3047 (2)	0.0468 (8)	
H26D	−0.0512	−0.0388	0.2990	0.070*	
H26E	−0.1756	−0.0211	0.3495	0.070*	
H26F	−0.0726	0.0441	0.3167	0.070*	
C27B	−0.1926 (3)	−0.02034 (17)	0.2077 (2)	0.0448 (7)	
H27D	−0.1431	−0.0667	0.2096	0.067*	
H27E	−0.2030	0.0034	0.1534	0.067*	
H27F	−0.2670	−0.0384	0.2489	0.067*	
C28B	0.1323 (2)	0.18389 (16)	−0.00721 (17)	0.0350 (6)	
H28B	0.0570	0.1768	0.0361	0.042*	
C29B	0.1096 (3)	0.15814 (19)	−0.07238 (19)	0.0461 (7)	
H29D	0.0549	0.1943	−0.0956	0.069*	
H29E	0.0780	0.1024	−0.0476	0.069*	
H29F	0.1814	0.1612	−0.1156	0.069*	
C30B	0.1611 (3)	0.27536 (17)	−0.0431 (2)	0.0472 (7)	
H30D	0.0976	0.3045	−0.0611	0.071*	
H30E	0.2306	0.2870	−0.0898	0.071*	
H30F	0.1734	0.2929	−0.0014	0.071*	
C31B	0.2215 (2)	0.01270 (16)	0.04315 (17)	0.0343 (6)	
H31B	0.2212	0.0222	−0.0137	0.041*	
C32B	0.1061 (3)	−0.02930 (17)	0.0982 (2)	0.0460 (7)	
H32D	0.0988	−0.0801	0.0900	0.069*	
H32E	0.0445	0.0061	0.0846	0.069*	
H32F	0.1010	−0.0408	0.1553	0.069*	
C33B	0.3175 (3)	−0.04613 (17)	0.0568 (2)	0.0453 (7)	
H33D	0.3105	−0.0909	0.0390	0.068*	
H33E	0.3117	−0.0675	0.1149	0.068*	
H33F	0.3917	−0.0176	0.0253	0.068*	
C34B	0.3898 (2)	0.15321 (16)	−0.00755 (17)	0.0345 (6)	
H34B	0.4361	0.1104	0.0206	0.041*	
C35B	0.4217 (3)	0.23240 (18)	−0.0014 (2)	0.0458 (7)	
H35D	0.5049	0.2415	−0.0223	0.069*	
H35E	0.3949	0.2286	0.0558	0.069*	
H35F	0.3856	0.2777	−0.0338	0.069*	
C36B	0.4272 (3)	0.15781 (19)	−0.09643 (18)	0.0465 (7)	
H36D	0.5100	0.1686	−0.1198	0.070*	
H36E	0.3877	0.2015	−0.1277	0.070*	
H36F	0.4077	0.1062	−0.0987	0.070*	

### Atomic displacement parameters (Å^2^)

**Table:**

	*U*^11^	*U*^22^	*U*^33^	*U*^12^	*U*^13^	*U*^23^
Ni1A	0.02734 (17)	0.02433 (16)	0.02715 (16)	−0.00333 (12)	−0.00748 (13)	−0.00737 (12)
Si1A	0.0262 (4)	0.0254 (3)	0.0295 (3)	−0.0019 (3)	−0.0103 (3)	−0.0081 (3)
Si2A	0.0282 (4)	0.0235 (3)	0.0263 (3)	−0.0022 (3)	−0.0077 (3)	−0.0076 (3)
N1A	0.0300 (11)	0.0262 (10)	0.0319 (11)	0.0023 (8)	−0.0117 (9)	−0.0103 (9)
N2A	0.0251 (11)	0.0252 (10)	0.0274 (10)	−0.0027 (8)	−0.0071 (8)	−0.0080 (8)
N3A	0.0294 (11)	0.0289 (11)	0.0325 (11)	−0.0006 (8)	−0.0123 (9)	−0.0110 (9)
N4A	0.0290 (11)	0.0246 (10)	0.0274 (10)	−0.0009 (8)	−0.0085 (9)	−0.0089 (8)
C1A	0.0366 (15)	0.0343 (14)	0.0360 (14)	0.0022 (11)	−0.0150 (12)	−0.0103 (11)
C2A	0.0346 (15)	0.0462 (16)	0.0482 (17)	0.0080 (12)	−0.0230 (13)	−0.0216 (14)
C3A	0.0270 (14)	0.0411 (15)	0.0504 (17)	0.0026 (11)	−0.0139 (12)	−0.0265 (13)
C4A	0.0265 (13)	0.0289 (12)	0.0378 (14)	0.0006 (10)	−0.0082 (11)	−0.0180 (11)
C5A	0.0251 (13)	0.0319 (13)	0.0446 (15)	−0.0025 (10)	−0.0041 (11)	−0.0183 (12)
C6A	0.0333 (14)	0.0293 (13)	0.0338 (14)	−0.0015 (11)	−0.0022 (11)	−0.0082 (11)
C7A	0.0284 (13)	0.0324 (13)	0.0305 (13)	0.0011 (10)	−0.0067 (11)	−0.0095 (11)
C8A	0.0250 (12)	0.0231 (11)	0.0328 (13)	0.0006 (9)	−0.0065 (10)	−0.0128 (10)
C9A	0.0268 (13)	0.0248 (12)	0.0324 (13)	0.0006 (9)	−0.0081 (10)	−0.0142 (10)
C10A	0.0325 (14)	0.0315 (13)	0.0447 (15)	−0.0021 (11)	−0.0154 (12)	−0.0132 (12)
C11A	0.0338 (15)	0.0329 (14)	0.0578 (18)	0.0034 (11)	−0.0204 (13)	−0.0238 (13)
C12A	0.0316 (14)	0.0428 (15)	0.0502 (17)	0.0085 (12)	−0.0191 (13)	−0.0284 (13)
C13A	0.0267 (13)	0.0379 (14)	0.0361 (14)	0.0045 (10)	−0.0153 (11)	−0.0174 (11)
C14A	0.0292 (14)	0.0470 (16)	0.0316 (14)	0.0048 (12)	−0.0070 (11)	−0.0168 (12)
C15A	0.0330 (15)	0.0429 (15)	0.0287 (13)	−0.0008 (12)	−0.0056 (11)	−0.0096 (11)
C16A	0.0343 (14)	0.0331 (13)	0.0305 (13)	−0.0009 (11)	−0.0085 (11)	−0.0111 (11)
C17A	0.0266 (13)	0.0292 (12)	0.0283 (12)	−0.0002 (10)	−0.0117 (10)	−0.0097 (10)
C18A	0.0261 (13)	0.0313 (12)	0.0297 (12)	0.0014 (10)	−0.0141 (10)	−0.0114 (10)
C19A	0.0313 (14)	0.0265 (12)	0.0363 (14)	0.0003 (10)	−0.0135 (11)	−0.0088 (10)
C20A	0.0475 (17)	0.0316 (14)	0.0480 (17)	0.0018 (12)	−0.0211 (14)	−0.0148 (12)
C21A	0.0397 (16)	0.0329 (14)	0.0496 (17)	0.0041 (12)	−0.0222 (13)	−0.0117 (12)
C22A	0.0374 (15)	0.0313 (13)	0.0334 (14)	0.0002 (11)	−0.0151 (12)	−0.0110 (11)
C23A	0.076 (2)	0.0461 (18)	0.0320 (15)	−0.0138 (16)	−0.0083 (15)	−0.0115 (13)
C24A	0.0406 (16)	0.0411 (15)	0.0406 (15)	0.0058 (12)	−0.0162 (13)	−0.0188 (13)
C25A	0.0279 (13)	0.0294 (13)	0.0345 (14)	−0.0017 (10)	−0.0104 (11)	−0.0068 (11)
C26A	0.0314 (15)	0.0434 (16)	0.0510 (17)	−0.0084 (12)	−0.0118 (13)	−0.0171 (14)
C27A	0.0302 (14)	0.0466 (16)	0.0377 (15)	−0.0037 (12)	−0.0075 (12)	−0.0118 (13)
C28A	0.0344 (14)	0.0246 (12)	0.0330 (13)	0.0005 (10)	−0.0077 (11)	−0.0115 (10)
C29A	0.0301 (15)	0.0388 (15)	0.0458 (16)	−0.0005 (11)	−0.0044 (12)	−0.0155 (13)
C30A	0.0430 (16)	0.0329 (14)	0.0309 (14)	−0.0004 (11)	−0.0083 (12)	−0.0107 (11)
C31A	0.0349 (15)	0.0330 (13)	0.0316 (13)	−0.0089 (11)	−0.0091 (11)	−0.0111 (11)
C32A	0.0377 (16)	0.0479 (17)	0.0546 (18)	−0.0028 (13)	−0.0213 (14)	−0.0191 (14)
C33A	0.0496 (18)	0.0385 (15)	0.0450 (16)	−0.0133 (13)	−0.0129 (14)	−0.0171 (13)
C34A	0.0385 (15)	0.0292 (13)	0.0319 (13)	−0.0007 (11)	−0.0120 (12)	−0.0064 (11)
C35A	0.070 (2)	0.0395 (16)	0.0576 (19)	0.0039 (15)	−0.0422 (18)	−0.0160 (14)
C36A	0.061 (2)	0.0297 (14)	0.0488 (17)	0.0057 (13)	−0.0256 (15)	−0.0084 (13)
Ni1B	0.02493 (17)	0.02550 (16)	0.03428 (18)	0.00398 (12)	−0.01139 (13)	−0.01210 (13)
Si1B	0.0247 (3)	0.0248 (3)	0.0355 (4)	0.0003 (3)	−0.0091 (3)	−0.0120 (3)
Si2B	0.0252 (3)	0.0255 (3)	0.0302 (3)	0.0000 (3)	−0.0065 (3)	−0.0104 (3)
N1B	0.0271 (11)	0.0304 (11)	0.0348 (11)	−0.0010 (8)	−0.0099 (9)	−0.0134 (9)
N2B	0.0260 (11)	0.0243 (10)	0.0354 (11)	0.0017 (8)	−0.0102 (9)	−0.0129 (9)
N3B	0.0269 (11)	0.0282 (10)	0.0327 (11)	−0.0006 (8)	−0.0092 (9)	−0.0125 (9)
N4B	0.0235 (10)	0.0261 (10)	0.0318 (11)	0.0029 (8)	−0.0090 (9)	−0.0108 (9)
C1B	0.0292 (14)	0.0394 (15)	0.0430 (15)	−0.0025 (11)	−0.0101 (12)	−0.0169 (12)
C2B	0.0399 (16)	0.0382 (15)	0.0449 (16)	−0.0097 (12)	−0.0110 (13)	−0.0148 (13)
C3B	0.0484 (18)	0.0278 (13)	0.0436 (16)	−0.0072 (12)	−0.0166 (13)	−0.0109 (12)
C4B	0.0429 (16)	0.0247 (12)	0.0339 (14)	0.0001 (11)	−0.0171 (12)	−0.0109 (10)
C5B	0.0498 (18)	0.0240 (12)	0.0529 (17)	0.0089 (12)	−0.0268 (14)	−0.0142 (12)
C6B	0.0356 (15)	0.0342 (14)	0.0555 (18)	0.0123 (11)	−0.0237 (13)	−0.0206 (13)
C7B	0.0270 (14)	0.0310 (13)	0.0492 (16)	0.0031 (10)	−0.0159 (12)	−0.0174 (12)
C8B	0.0303 (13)	0.0251 (12)	0.0335 (13)	0.0021 (10)	−0.0135 (11)	−0.0110 (10)
C9B	0.0303 (13)	0.0285 (12)	0.0312 (13)	0.0014 (10)	−0.0139 (11)	−0.0118 (10)
C10B	0.0321 (15)	0.0383 (14)	0.0406 (15)	0.0012 (11)	−0.0086 (12)	−0.0198 (12)
C11B	0.0423 (16)	0.0433 (15)	0.0340 (14)	−0.0049 (12)	−0.0086 (12)	−0.0178 (12)
C12B	0.0437 (17)	0.0385 (15)	0.0330 (14)	−0.0084 (12)	−0.0154 (12)	−0.0079 (12)
C13B	0.0307 (14)	0.0300 (13)	0.0338 (13)	−0.0042 (10)	−0.0129 (11)	−0.0083 (11)
C14B	0.0362 (15)	0.0360 (14)	0.0412 (16)	0.0043 (11)	−0.0200 (13)	−0.0066 (12)
C15B	0.0316 (15)	0.0374 (15)	0.0522 (18)	0.0095 (12)	−0.0183 (13)	−0.0119 (13)
C16B	0.0275 (13)	0.0349 (14)	0.0376 (14)	0.0042 (10)	−0.0094 (11)	−0.0133 (11)
C17B	0.0249 (12)	0.0233 (11)	0.0334 (13)	−0.0027 (9)	−0.0101 (10)	−0.0096 (10)
C18B	0.0255 (12)	0.0251 (12)	0.0339 (13)	−0.0033 (9)	−0.0118 (10)	−0.0096 (10)
C19B	0.0282 (14)	0.0347 (14)	0.0391 (15)	0.0012 (11)	−0.0058 (11)	−0.0136 (12)
C20B	0.0460 (18)	0.0536 (18)	0.0423 (17)	0.0011 (14)	−0.0099 (14)	−0.0241 (14)
C21B	0.0375 (16)	0.0423 (16)	0.0493 (18)	−0.0074 (13)	−0.0020 (13)	−0.0134 (14)
C22B	0.0330 (15)	0.0350 (14)	0.0449 (16)	−0.0003 (11)	−0.0170 (12)	−0.0154 (12)
C23B	0.0471 (19)	0.070 (2)	0.0388 (16)	−0.0035 (16)	−0.0153 (14)	−0.0155 (15)
C24B	0.0363 (16)	0.0483 (17)	0.0584 (19)	0.0009 (13)	−0.0214 (14)	−0.0261 (15)
C25B	0.0306 (14)	0.0247 (12)	0.0505 (17)	0.0005 (10)	−0.0072 (12)	−0.0137 (12)
C26B	0.0405 (17)	0.0289 (14)	0.068 (2)	0.0029 (12)	−0.0220 (15)	−0.0092 (14)
C27B	0.0430 (17)	0.0291 (14)	0.062 (2)	0.0000 (12)	−0.0094 (15)	−0.0204 (13)
C28B	0.0348 (15)	0.0376 (14)	0.0333 (14)	0.0037 (11)	−0.0116 (11)	−0.0126 (11)
C29B	0.0547 (19)	0.0499 (17)	0.0411 (16)	0.0024 (14)	−0.0241 (15)	−0.0166 (14)
C30B	0.059 (2)	0.0357 (15)	0.0495 (18)	0.0107 (14)	−0.0252 (15)	−0.0118 (13)
C31B	0.0318 (14)	0.0319 (13)	0.0426 (15)	0.0007 (11)	−0.0087 (12)	−0.0190 (12)
C32B	0.0364 (16)	0.0350 (15)	0.066 (2)	−0.0039 (12)	−0.0061 (14)	−0.0237 (14)
C33B	0.0395 (17)	0.0351 (15)	0.066 (2)	0.0051 (12)	−0.0124 (15)	−0.0270 (14)
C34B	0.0302 (14)	0.0318 (13)	0.0367 (14)	−0.0034 (11)	−0.0048 (11)	−0.0100 (11)
C35B	0.0343 (16)	0.0451 (17)	0.0565 (19)	−0.0094 (13)	−0.0112 (14)	−0.0162 (14)
C36B	0.0413 (17)	0.0463 (17)	0.0400 (16)	−0.0031 (13)	0.0011 (13)	−0.0116 (13)

### Geometric parameters (Å, °)

**Table:**

Ni1A—N4A	1.955 (2)	Ni1B—N4B	1.960 (2)
Ni1A—N2A	1.977 (2)	Ni1B—N2B	1.962 (2)
Ni1A—N1A	2.002 (2)	Ni1B—N3B	2.002 (2)
Ni1A—N3A	2.001 (2)	Ni1B—N1B	2.005 (2)
Si1A—N2A	1.754 (2)	Si1B—N2B	1.744 (2)
Si1A—C22A	1.906 (3)	Si1B—C25B	1.892 (3)
Si1A—C19A	1.905 (3)	Si1B—C22B	1.903 (3)
Si1A—C25A	1.907 (3)	Si1B—C19B	1.903 (3)
Si2A—N4A	1.755 (2)	Si2B—N4B	1.745 (2)
Si2A—C28A	1.876 (3)	Si2B—C34B	1.900 (3)
Si2A—C31A	1.909 (3)	Si2B—C31B	1.906 (3)
Si2A—C34A	1.906 (3)	Si2B—C28B	1.908 (3)
N1A—C1A	1.328 (3)	N1B—C1B	1.321 (3)
N1A—C9A	1.365 (3)	N1B—C9B	1.365 (3)
N2A—C8A	1.369 (3)	N2B—C8B	1.374 (3)
N3A—C10A	1.324 (3)	N3B—C10B	1.319 (3)
N3A—C18A	1.366 (3)	N3B—C18B	1.369 (3)
N4A—C17A	1.375 (3)	N4B—C17B	1.373 (3)
C1A—C2A	1.401 (4)	C1B—C2B	1.395 (4)
C1A—H1AA	0.9500	C1B—H1BA	0.9500
C2A—C3A	1.368 (4)	C2B—C3B	1.367 (4)
C2A—H2AA	0.9500	C2B—H2BA	0.9500
C3A—C4A	1.411 (4)	C3B—C4B	1.412 (4)
C3A—H3AA	0.9500	C3B—H3BA	0.9500
C4A—C9A	1.413 (4)	C4B—C5B	1.413 (4)
C4A—C5A	1.412 (4)	C4B—C9B	1.417 (3)
C5A—C6A	1.372 (4)	C5B—C6B	1.369 (4)
C5A—H5AA	0.9500	C5B—H5BA	0.9500
C6A—C7A	1.407 (4)	C6B—C7B	1.408 (4)
C6A—H6AA	0.9500	C6B—H6BA	0.9500
C7A—C8A	1.395 (3)	C7B—C8B	1.400 (4)
C7A—H7AA	0.9500	C7B—H7BA	0.9500
C8A—C9A	1.451 (3)	C8B—C9B	1.440 (4)
C10A—C11A	1.404 (4)	C10B—C11B	1.400 (4)
C10A—H10A	0.9500	C10B—H10B	0.9500
C11A—C12A	1.364 (4)	C11B—C12B	1.367 (4)
C11A—H11A	0.9500	C11B—H11B	0.9500
C12A—C13A	1.421 (4)	C12B—C13B	1.410 (4)
C12A—H12A	0.9500	C12B—H12B	0.9500
C13A—C14A	1.408 (4)	C13B—C14B	1.417 (4)
C13A—C18A	1.412 (4)	C13B—C18B	1.421 (3)
C14A—C15A	1.370 (4)	C14B—C15B	1.365 (4)
C14A—H14A	0.9500	C14B—H14B	0.9500
C15A—C16A	1.403 (4)	C15B—C16B	1.402 (4)
C15A—H15A	0.9500	C15B—H15B	0.9500
C16A—C17A	1.398 (4)	C16B—C17B	1.405 (3)
C16A—H16A	0.9500	C16B—H16B	0.9500
C17A—C18A	1.438 (3)	C17B—C18B	1.437 (4)
C19A—C20A	1.534 (4)	C19B—C21B	1.533 (4)
C19A—C21A	1.541 (4)	C19B—C20B	1.538 (4)
C19A—H19A	1.0000	C19B—H19B	1.0000
C20A—H20A	0.9800	C20B—H20D	0.9800
C20A—H20B	0.9800	C20B—H20E	0.9800
C20A—H20C	0.9800	C20B—H20F	0.9800
C21A—H21A	0.9800	C21B—H21D	0.9800
C21A—H21B	0.9800	C21B—H21E	0.9800
C21A—H21C	0.9800	C21B—H21F	0.9800
C22A—C24A	1.522 (4)	C22B—C23B	1.533 (4)
C22A—C23A	1.529 (4)	C22B—C24B	1.539 (4)
C22A—H22A	1.0000	C22B—H22B	1.0000
C23A—H23A	0.9800	C23B—H23D	0.9800
C23A—H23B	0.9800	C23B—H23E	0.9800
C23A—H23C	0.9800	C23B—H23F	0.9800
C24A—H24A	0.9800	C24B—H24D	0.9800
C24A—H24B	0.9800	C24B—H24E	0.9800
C24A—H24C	0.9800	C24B—H24F	0.9800
C25A—C27A	1.536 (4)	C25B—C27B	1.538 (4)
C25A—C26A	1.543 (4)	C25B—C26B	1.535 (4)
C25A—H25A	1.0000	C25B—H25B	1.0000
C26A—H26A	0.9800	C26B—H26D	0.9800
C26A—H26B	0.9800	C26B—H26E	0.9800
C26A—H26C	0.9800	C26B—H26F	0.9800
C27A—H27A	0.9800	C27B—H27D	0.9800
C27A—H27B	0.9800	C27B—H27E	0.9800
C27A—H27C	0.9800	C27B—H27F	0.9800
C28A—C29A	1.533 (4)	C28B—C29B	1.532 (4)
C28A—C30A	1.534 (4)	C28B—C30B	1.536 (4)
C28A—H28A	1.0000	C28B—H28B	1.0000
C29A—H29A	0.9800	C29B—H29D	0.9800
C29A—H29B	0.9800	C29B—H29E	0.9800
C29A—H29C	0.9800	C29B—H29F	0.9800
C30A—H30A	0.9800	C30B—H30D	0.9800
C30A—H30B	0.9800	C30B—H30E	0.9800
C30A—H30C	0.9800	C30B—H30F	0.9800
C31A—C32A	1.538 (4)	C31B—C32B	1.527 (4)
C31A—C33A	1.544 (4)	C31B—C33B	1.532 (4)
C31A—H31A	1.0000	C31B—H31B	1.0000
C32A—H32A	0.9800	C32B—H32D	0.9800
C32A—H32B	0.9800	C32B—H32E	0.9800
C32A—H32C	0.9800	C32B—H32F	0.9800
C33A—H33A	0.9800	C33B—H33D	0.9800
C33A—H33B	0.9800	C33B—H33E	0.9800
C33A—H33C	0.9800	C33B—H33F	0.9800
C34A—C36A	1.532 (4)	C34B—C36B	1.531 (4)
C34A—C35A	1.535 (4)	C34B—C35B	1.536 (4)
C34A—H34A	1.0000	C34B—H34B	1.0000
C35A—H35A	0.9800	C35B—H35D	0.9800
C35A—H35B	0.9800	C35B—H35E	0.9800
C35A—H35C	0.9800	C35B—H35F	0.9800
C36A—H36A	0.9800	C36B—H36D	0.9800
C36A—H36B	0.9800	C36B—H36E	0.9800
C36A—H36C	0.9800	C36B—H36F	0.9800
			
N4A—Ni1A—N2A	148.45 (9)	N4B—Ni1B—N2B	148.84 (9)
N4A—Ni1A—N1A	122.96 (8)	N4B—Ni1B—N3B	84.27 (9)
N2A—Ni1A—N1A	84.10 (8)	N2B—Ni1B—N3B	112.36 (9)
N4A—Ni1A—N3A	84.62 (9)	N4B—Ni1B—N1B	118.98 (9)
N2A—Ni1A—N3A	109.19 (8)	N2B—Ni1B—N1B	83.97 (8)
N1A—Ni1A—N3A	98.12 (9)	N3B—Ni1B—N1B	103.14 (8)
N2A—Si1A—C22A	116.87 (11)	N2B—Si1B—C25B	104.23 (11)
N2A—Si1A—C19A	111.91 (11)	N2B—Si1B—C22B	108.52 (11)
C22A—Si1A—C19A	105.25 (11)	C25B—Si1B—C22B	111.75 (13)
N2A—Si1A—C25A	104.61 (10)	N2B—Si1B—C19B	112.00 (12)
C22A—Si1A—C25A	106.44 (12)	C25B—Si1B—C19B	111.95 (12)
C19A—Si1A—C25A	111.76 (12)	C22B—Si1B—C19B	108.35 (13)
N4A—Si2A—C28A	109.15 (10)	N4B—Si2B—C34B	112.65 (11)
N4A—Si2A—C31A	111.73 (11)	N4B—Si2B—C31B	114.47 (11)
C28A—Si2A—C31A	108.86 (12)	C34B—Si2B—C31B	106.42 (12)
N4A—Si2A—C34A	110.77 (11)	N4B—Si2B—C28B	103.96 (11)
C28A—Si2A—C34A	109.65 (12)	C34B—Si2B—C28B	112.80 (12)
C31A—Si2A—C34A	106.62 (12)	C31B—Si2B—C28B	106.53 (12)
C1A—N1A—C9A	119.8 (2)	C1B—N1B—C9B	119.4 (2)
C1A—N1A—Ni1A	128.39 (18)	C1B—N1B—Ni1B	129.09 (18)
C9A—N1A—Ni1A	111.79 (16)	C9B—N1B—Ni1B	111.53 (16)
C8A—N2A—Si1A	125.42 (17)	C8B—N2B—Si1B	124.02 (17)
C8A—N2A—Ni1A	111.27 (16)	C8B—N2B—Ni1B	111.78 (16)
Si1A—N2A—Ni1A	122.92 (11)	Si1B—N2B—Ni1B	124.11 (11)
C10A—N3A—C18A	119.9 (2)	C10B—N3B—C18B	119.7 (2)
C10A—N3A—Ni1A	128.79 (19)	C10B—N3B—Ni1B	128.84 (18)
C18A—N3A—Ni1A	110.72 (16)	C18B—N3B—Ni1B	111.30 (16)
C17A—N4A—Si2A	120.87 (16)	C17B—N4B—Si2B	125.04 (17)
C17A—N4A—Ni1A	110.75 (15)	C17B—N4B—Ni1B	111.40 (16)
Si2A—N4A—Ni1A	128.38 (12)	Si2B—N4B—Ni1B	123.53 (11)
N1A—C1A—C2A	122.4 (3)	N1B—C1B—C2B	122.9 (3)
N1A—C1A—H1AA	118.8	N1B—C1B—H1BA	118.6
C2A—C1A—H1AA	118.8	C2B—C1B—H1BA	118.6
C3A—C2A—C1A	118.5 (2)	C3B—C2B—C1B	118.8 (3)
C3A—C2A—H2AA	120.7	C3B—C2B—H2BA	120.6
C1A—C2A—H2AA	120.7	C1B—C2B—H2BA	120.6
C2A—C3A—C4A	120.9 (3)	C2B—C3B—C4B	120.4 (2)
C2A—C3A—H3AA	119.6	C2B—C3B—H3BA	119.8
C4A—C3A—H3AA	119.6	C4B—C3B—H3BA	119.8
C3A—C4A—C9A	117.1 (2)	C3B—C4B—C5B	124.2 (2)
C3A—C4A—C5A	124.1 (2)	C3B—C4B—C9B	117.0 (2)
C9A—C4A—C5A	118.8 (2)	C5B—C4B—C9B	118.8 (2)
C6A—C5A—C4A	118.7 (2)	C6B—C5B—C4B	118.8 (2)
C6A—C5A—H5AA	120.6	C6B—C5B—H5BA	120.6
C4A—C5A—H5AA	120.6	C4B—C5B—H5BA	120.6
C5A—C6A—C7A	122.3 (2)	C5B—C6B—C7B	122.4 (2)
C5A—C6A—H6AA	118.9	C5B—C6B—H6BA	118.8
C7A—C6A—H6AA	118.9	C7B—C6B—H6BA	118.8
C8A—C7A—C6A	122.6 (2)	C8B—C7B—C6B	121.9 (2)
C8A—C7A—H7AA	118.7	C8B—C7B—H7BA	119.1
C6A—C7A—H7AA	118.7	C6B—C7B—H7BA	119.1
N2A—C8A—C7A	128.3 (2)	N2B—C8B—C7B	128.0 (2)
N2A—C8A—C9A	117.4 (2)	N2B—C8B—C9B	117.0 (2)
C7A—C8A—C9A	114.3 (2)	C7B—C8B—C9B	115.0 (2)
N1A—C9A—C4A	121.4 (2)	N1B—C9B—C4B	121.5 (2)
N1A—C9A—C8A	115.3 (2)	N1B—C9B—C8B	115.6 (2)
C4A—C9A—C8A	123.3 (2)	C4B—C9B—C8B	123.0 (2)
N3A—C10A—C11A	122.1 (3)	N3B—C10B—C11B	122.5 (3)
N3A—C10A—H10A	119.0	N3B—C10B—H10B	118.8
C11A—C10A—H10A	119.0	C11B—C10B—H10B	118.8
C12A—C11A—C10A	119.1 (2)	C12B—C11B—C10B	119.2 (3)
C12A—C11A—H11A	120.5	C12B—C11B—H11B	120.4
C10A—C11A—H11A	120.5	C10B—C11B—H11B	120.4
C11A—C12A—C13A	120.6 (3)	C11B—C12B—C13B	120.2 (3)
C11A—C12A—H12A	119.7	C11B—C12B—H12B	119.9
C13A—C12A—H12A	119.7	C13B—C12B—H12B	119.9
C14A—C13A—C18A	119.1 (2)	C12B—C13B—C14B	124.3 (2)
C14A—C13A—C12A	124.2 (3)	C12B—C13B—C18B	117.2 (2)
C18A—C13A—C12A	116.7 (2)	C14B—C13B—C18B	118.4 (2)
C15A—C14A—C13A	118.6 (3)	C15B—C14B—C13B	118.5 (2)
C15A—C14A—H14A	120.7	C15B—C14B—H14B	120.7
C13A—C14A—H14A	120.7	C13B—C14B—H14B	120.7
C14A—C15A—C16A	122.3 (3)	C14B—C15B—C16B	123.1 (3)
C14A—C15A—H15A	118.9	C14B—C15B—H15B	118.4
C16A—C15A—H15A	118.9	C16B—C15B—H15B	118.4
C17A—C16A—C15A	122.2 (2)	C15B—C16B—C17B	121.7 (3)
C17A—C16A—H16A	118.9	C15B—C16B—H16B	119.1
C15A—C16A—H16A	118.9	C17B—C16B—H16B	119.1
N4A—C17A—C16A	127.5 (2)	N4B—C17B—C16B	127.8 (2)
N4A—C17A—C18A	117.7 (2)	N4B—C17B—C18B	117.5 (2)
C16A—C17A—C18A	114.8 (2)	C16B—C17B—C18B	114.7 (2)
N3A—C18A—C13A	121.7 (2)	N3B—C18B—C13B	121.2 (2)
N3A—C18A—C17A	115.4 (2)	N3B—C18B—C17B	115.3 (2)
C13A—C18A—C17A	122.9 (2)	C13B—C18B—C17B	123.4 (2)
C20A—C19A—C21A	111.7 (2)	C21B—C19B—C20B	110.4 (2)
C20A—C19A—Si1A	113.78 (18)	C21B—C19B—Si1B	113.8 (2)
C21A—C19A—Si1A	112.23 (18)	C20B—C19B—Si1B	111.94 (19)
C20A—C19A—H19A	106.2	C21B—C19B—H19B	106.8
C21A—C19A—H19A	106.2	C20B—C19B—H19B	106.8
Si1A—C19A—H19A	106.2	Si1B—C19B—H19B	106.8
C19A—C20A—H20A	109.5	C19B—C20B—H20D	109.5
C19A—C20A—H20B	109.5	C19B—C20B—H20E	109.5
H20A—C20A—H20B	109.5	H20D—C20B—H20E	109.5
C19A—C20A—H20C	109.5	C19B—C20B—H20F	109.5
H20A—C20A—H20C	109.5	H20D—C20B—H20F	109.5
H20B—C20A—H20C	109.5	H20E—C20B—H20F	109.5
C19A—C21A—H21A	109.5	C19B—C21B—H21D	109.5
C19A—C21A—H21B	109.5	C19B—C21B—H21E	109.5
H21A—C21A—H21B	109.5	H21D—C21B—H21E	109.5
C19A—C21A—H21C	109.5	C19B—C21B—H21F	109.5
H21A—C21A—H21C	109.5	H21D—C21B—H21F	109.5
H21B—C21A—H21C	109.5	H21E—C21B—H21F	109.5
C24A—C22A—C23A	108.8 (2)	C23B—C22B—C24B	110.4 (2)
C24A—C22A—Si1A	114.80 (18)	C23B—C22B—Si1B	112.7 (2)
C23A—C22A—Si1A	117.6 (2)	C24B—C22B—Si1B	116.8 (2)
C24A—C22A—H22A	104.8	C23B—C22B—H22B	105.3
C23A—C22A—H22A	104.8	C24B—C22B—H22B	105.3
Si1A—C22A—H22A	104.8	Si1B—C22B—H22B	105.3
C22A—C23A—H23A	109.5	C22B—C23B—H23D	109.5
C22A—C23A—H23B	109.5	C22B—C23B—H23E	109.5
H23A—C23A—H23B	109.5	H23D—C23B—H23E	109.5
C22A—C23A—H23C	109.5	C22B—C23B—H23F	109.5
H23A—C23A—H23C	109.5	H23D—C23B—H23F	109.5
H23B—C23A—H23C	109.5	H23E—C23B—H23F	109.5
C22A—C24A—H24A	109.5	C22B—C24B—H24D	109.5
C22A—C24A—H24B	109.5	C22B—C24B—H24E	109.5
H24A—C24A—H24B	109.5	H24D—C24B—H24E	109.5
C22A—C24A—H24C	109.5	C22B—C24B—H24F	109.5
H24A—C24A—H24C	109.5	H24D—C24B—H24F	109.5
H24B—C24A—H24C	109.5	H24E—C24B—H24F	109.5
C27A—C25A—C26A	107.4 (2)	C27B—C25B—C26B	109.3 (2)
C27A—C25A—Si1A	115.79 (18)	C27B—C25B—Si1B	116.5 (2)
C26A—C25A—Si1A	115.25 (19)	C26B—C25B—Si1B	115.1 (2)
C27A—C25A—H25A	105.8	C27B—C25B—H25B	104.9
C26A—C25A—H25A	105.8	C26B—C25B—H25B	104.9
Si1A—C25A—H25A	105.8	Si1B—C25B—H25B	104.9
C25A—C26A—H26A	109.5	C25B—C26B—H26D	109.5
C25A—C26A—H26B	109.5	C25B—C26B—H26E	109.5
H26A—C26A—H26B	109.5	H26D—C26B—H26E	109.5
C25A—C26A—H26C	109.5	C25B—C26B—H26F	109.5
H26A—C26A—H26C	109.5	H26D—C26B—H26F	109.5
H26B—C26A—H26C	109.5	H26E—C26B—H26F	109.5
C25A—C27A—H27A	109.5	C25B—C27B—H27D	109.5
C25A—C27A—H27B	109.5	C25B—C27B—H27E	109.5
H27A—C27A—H27B	109.5	H27D—C27B—H27E	109.5
C25A—C27A—H27C	109.5	C25B—C27B—H27F	109.5
H27A—C27A—H27C	109.5	H27D—C27B—H27F	109.5
H27B—C27A—H27C	109.5	H27E—C27B—H27F	109.5
C29A—C28A—C30A	109.8 (2)	C29B—C28B—C30B	108.7 (2)
C29A—C28A—Si2A	115.17 (19)	C29B—C28B—Si2B	115.1 (2)
C30A—C28A—Si2A	112.65 (18)	C30B—C28B—Si2B	115.7 (2)
C29A—C28A—H28A	106.2	C29B—C28B—H28B	105.4
C30A—C28A—H28A	106.2	C30B—C28B—H28B	105.4
Si2A—C28A—H28A	106.2	Si2B—C28B—H28B	105.4
C28A—C29A—H29A	109.5	C28B—C29B—H29D	109.5
C28A—C29A—H29B	109.5	C28B—C29B—H29E	109.5
H29A—C29A—H29B	109.5	H29D—C29B—H29E	109.5
C28A—C29A—H29C	109.5	C28B—C29B—H29F	109.5
H29A—C29A—H29C	109.5	H29D—C29B—H29F	109.5
H29B—C29A—H29C	109.5	H29E—C29B—H29F	109.5
C28A—C30A—H30A	109.5	C28B—C30B—H30D	109.5
C28A—C30A—H30B	109.5	C28B—C30B—H30E	109.5
H30A—C30A—H30B	109.5	H30D—C30B—H30E	109.5
C28A—C30A—H30C	109.5	C28B—C30B—H30F	109.5
H30A—C30A—H30C	109.5	H30D—C30B—H30F	109.5
H30B—C30A—H30C	109.5	H30E—C30B—H30F	109.5
C32A—C31A—C33A	110.2 (2)	C32B—C31B—C33B	109.7 (2)
C32A—C31A—Si2A	114.30 (18)	C32B—C31B—Si2B	111.68 (19)
C33A—C31A—Si2A	114.0 (2)	C33B—C31B—Si2B	118.6 (2)
C32A—C31A—H31A	105.9	C32B—C31B—H31B	105.3
C33A—C31A—H31A	105.9	C33B—C31B—H31B	105.3
Si2A—C31A—H31A	105.9	Si2B—C31B—H31B	105.3
C31A—C32A—H32A	109.5	C31B—C32B—H32D	109.5
C31A—C32A—H32B	109.5	C31B—C32B—H32E	109.5
H32A—C32A—H32B	109.5	H32D—C32B—H32E	109.5
C31A—C32A—H32C	109.5	C31B—C32B—H32F	109.5
H32A—C32A—H32C	109.5	H32D—C32B—H32F	109.5
H32B—C32A—H32C	109.5	H32E—C32B—H32F	109.5
C31A—C33A—H33A	109.5	C31B—C33B—H33D	109.5
C31A—C33A—H33B	109.5	C31B—C33B—H33E	109.5
H33A—C33A—H33B	109.5	H33D—C33B—H33E	109.5
C31A—C33A—H33C	109.5	C31B—C33B—H33F	109.5
H33A—C33A—H33C	109.5	H33D—C33B—H33F	109.5
H33B—C33A—H33C	109.5	H33E—C33B—H33F	109.5
C36A—C34A—C35A	109.9 (2)	C36B—C34B—C35B	111.0 (2)
C36A—C34A—Si2A	113.1 (2)	C36B—C34B—Si2B	112.1 (2)
C35A—C34A—Si2A	113.44 (18)	C35B—C34B—Si2B	114.5 (2)
C36A—C34A—H34A	106.6	C36B—C34B—H34B	106.2
C35A—C34A—H34A	106.6	C35B—C34B—H34B	106.2
Si2A—C34A—H34A	106.6	Si2B—C34B—H34B	106.2
C34A—C35A—H35A	109.5	C34B—C35B—H35D	109.5
C34A—C35A—H35B	109.5	C34B—C35B—H35E	109.5
H35A—C35A—H35B	109.5	H35D—C35B—H35E	109.5
C34A—C35A—H35C	109.5	C34B—C35B—H35F	109.5
H35A—C35A—H35C	109.5	H35D—C35B—H35F	109.5
H35B—C35A—H35C	109.5	H35E—C35B—H35F	109.5
C34A—C36A—H36A	109.5	C34B—C36B—H36D	109.5
C34A—C36A—H36B	109.5	C34B—C36B—H36E	109.5
H36A—C36A—H36B	109.5	H36D—C36B—H36E	109.5
C34A—C36A—H36C	109.5	C34B—C36B—H36F	109.5
H36A—C36A—H36C	109.5	H36D—C36B—H36F	109.5
H36B—C36A—H36C	109.5	H36E—C36B—H36F	109.5
